# Timecourse of mirror and counter-mirror effects measured with transcranial magnetic stimulation

**DOI:** 10.1093/scan/nst085

**Published:** 2013-05-23

**Authors:** Andrea Cavallo, Cecilia Heyes, Cristina Becchio, Geoffrey Bird, Caroline Catmur

**Affiliations:** ^1^Università di Torino, Dipartimento di Psicologia, Centro di Scienza Cognitiva, Turin, Italy, ^2^All Souls College, University of Oxford, Oxford, OX1 4AL, UK, ^3^Department of Experimental Psychology, University of Oxford, Oxford OX1 3UD, UK, ^4^MRC Social, Genetic & Developmental Psychiatry Centre, Institute of Psychiatry, Kings College London, London SE5 8AF, UK, and ^5^Department of Psychology, University of Surrey, Guildford GU2 7XH, UK

**Keywords:** mirror neuron, mirror neuron system, transcranial magnetic stimulation, sensorimotor learning, timecourse

## Abstract

The human mirror system has been the subject of much research over the past two decades, but little is known about the timecourse of mirror responses. In addition, it is unclear whether mirror and counter-mirror effects follow the same timecourse. We used single-pulse transcranial magnetic stimulation to investigate the timecourse of mirror and counter-mirror responses in the human brain. Experiment 1 demonstrated that mirror responses can be measured from around 200 ms after observed action onset. Experiment 2 demonstrated significant effects of counter-mirror sensorimotor training at all timepoints at which a mirror response was found in Experiment 1 (i.e. from 200 ms onward), indicating that mirror and counter-mirror responses follow the same timecourse. By suggesting similarly direct routes for mirror and counter-mirror responses, these results support the associative account of mirror neuron origins whereby mirror responses arise as a result of correlated sensorimotor experience during development. More generally, they contribute to theorizing regarding mirror neuron function by providing some constraints on how quickly mirror responses can influence social cognition.

## INTRODUCTION

Mirror neurons, which fire both when performing an action and when observing another performing the same action, have been the focus of much interest and speculation since their discovery in the macaque (di Pellegrino *et al.*, 1992). Converging evidence using a range of techniques suggests that these neurons are also present in the human brain (Fadiga *et al.*, 1995; Nishitani and Hari, 2000; Oberman *et al.*, 2007; Borroni *et al.*, 2008; Etzel *et al.*, 2008; Keysers and Gazzola, 2009; Kilner *et al.*, 2009; Mukamel *et al.*, 2010; Oosterhof *et al.*, 2010, 2012). The original explanation for mirror neurons’ fascinating response properties was that they have evolved to allow ‘action understanding’—the ability to use one’s own motor representations to simulate another agent’s actions and hence gain insight into their intentions (Gallese *et al.*, 1996). Other explanations have also been proposed, including the possibility that mirror response properties arise as a result of sensorimotor experience gained during development (Heyes, 2001, 2010; Keysers and Perrett, 2004). The latter explanation does not deny that mirror neurons may contribute to social interaction in important ways, but emphasizes the role of experiential and cultural factors in the formation of their response properties.

Due to the difficulty of recording from single neurons in the intact human brain, a variety of methods have been used to measure ‘mirror’ responses to observation of others’ actions. Standard functional magnetic resonance imaging (fMRI) techniques can identify, with high anatomical precision, the areas involved in action execution which respond to action observation. However, these techniques cannot identify whether such responses correspond to activation of a motor program that *matches* the observed action; operationally, it is this matching property that defines mirror responses. In contrast, methods that can determine the relative activity of specific motor programs during action observation have the potential to provide *operational* specificity. Such methods include the fMRI techniques of repetition suppression (Kilner *et al.*, 2009) and multi-voxel pattern analysis (Etzel *et al.*, 2008; Oosterhof *et al.*, 2010, 2012); the measurement of motor-evoked potentials (MEPs; Fadiga *et al.*, 1995) and evoked movements (Stefan *et al.*, 2005) using transcranial magnetic stimulation (TMS) and behavioural measurement of the extent to which observed actions interfere with action performance (Stürmer *et al.*, 2000; Kilner *et al.*, 2003). These methods involve different levels of neuroanatomical specificity, but provide operationally specific measurement of mirror responses, that is, the activation of motor programs matching observed actions.

One aspect of mirror responses which has so far received little attention is their timecourse: the length of time it takes for an observed action to activate a matching motor program. Investigation of the timecourse of mirror responses is of interest because—whether mirror responses are involved in understanding others’ actions or in other social cognitive processes—the timecourse of mirror responses places constraints on how quickly these processes can occur following the observation of another’s action. In addition, it has been proposed that timecourse information could help determine whether mirror activity is occurring via a more or less direct route from perceptual to motor areas (Barchiesi and Cattaneo, 2012).

### Timecourse of mirror responses

In order to assess the timecourse of mirror responses, it is important to use discrete, non-recurring actions such that the time of action onset can be clearly determined, and the action cannot be predicted in advance of its onset. To the extent that these conditions are met in the macaque mirror neuron literature, it is possible to estimate the timecourse of mirror neuron responses to perceived actions. It is clear that in premotor area F5 this timecourse varies widely (response latencies between 200 and 900 ms have been reported for visual stimuli; see Supplementary Data), depending on the stimulus type and task demands. Thus, the macaque mirror neuron literature does not currently provide a clear indication of how quickly mirror responses to others’ actions occur.

In humans, this question has been investigated using electroencephalography (EEG) and magnetoencephalography (MEG), which provide better temporal resolution than functional magnetic resonance imaging (fMRI). Single-pulse TMS can also provide useful information about the timing of neural responses. For example, early (90 ms) effects of action observation were demonstrated using TMS-evoked MEPs (Lepage *et al.*, 2010). MEPs recorded 90 ms after the onset of an observed index finger movement were greater than MEPs recorded during observation of a static hand or a moving dot. Critically, however, these effects were not muscle-specific: they were found in both the index and little finger muscles, regardless of whether these muscles would be involved in the observed action. Similar early non-specific effects of action observation were found using MEG at around 83 ms (van Schie *et al.*, 2008). In this case, participants could predict the likely observed action on the basis of a cue 400 ms before the action; prediction is known to modulate motor responses to observed actions up to 500 ms before action onset (Kilner *et al.*, 2004), and thus action prediction could contribute to the fast timecourse of responses to the observed action. Interestingly, the MEG response at 83 ms after action onset distinguished correct from incorrect observed actions on the basis of the side of space of the hand performing the action, but did not distinguish correct from incorrect goal location. Thus, this effect appears to reflect a fast response to whether or not the observed action is on the predicted side of space (see also Press *et al.*, 2010). Since the early effects described above provide minimal information about the identity of the observed action, they are likely not to be mirror effects but instead either more general alerting effects (e.g. due to the presence of a salient stimulus) or spatial compatibility effects. Spatial compatibility effects, in which a stimulus presented in one part of space facilitates a non-specific motor response at the same location (Simon, 1969), cannot be regarded as mirror responses because they do not reflect information about the identity (e.g. grip type, effector) of the observed action.

Valuable information about the potential timecourse and anatomical pathway of mirror responses has been provided by two MEG studies (Nishitani and Hari, 2000, 2002). These data indicate that information about observed actions is transmitted from visual to motor areas via superior temporal, parietal and premotor cortex, and that this process takes around 300 ms. However, these data cannot show whether the sight of an action activates a *matching* (i.e. mirror) motor representation or whether instead the observation of an action produces more general, non-specific motor responses. An alternative measure of mirror responses is therefore needed.

### Using MEPs to index muscle-specific mirror effects

Applying a TMS pulse to the primary motor cortex representation of a muscle produces an MEP in that muscle. Action observation induces changes in MEP size that are specific to the muscle that would be involved in the observed action (Fadiga *et al.*, 1995; Strafella and Paus, 2000). Thus unlike EEG, MEG and most fMRI measures, MEPs recorded during action observation index the *matching* properties of mirror neurons: the observation of an action produces effects on a measure of motor system activity that is specific to that action. Important information regarding the modulation of mirror responses during ongoing actions has been gained through measurement of MEPs (e.g. Gangitano *et al.*, 2001, 2004; Borroni *et al.*, 2011). However, since these studies were not designed to measure mirror response latency, the earliest timepoints used were 500 ms after the onset of the action, and therefore it is possible that mirror responses may occur earlier than this. In addition, these studies and others (e.g. Barchiesi and Cattaneo, 2012) used actions that gradually unfolded over time. Thus, they may have recruited predictive processes which, while important in action observation, would obscure information about mirror response timecourse, the focal issue in this study.

It is important to ensure that MEP responses are specific to the observed action *and* to the muscle that would perform that action. Otherwise, illusory ‘mirror’ responses could arise. For example, one muscle might display MEP enhancement in response to observation of the action in which it is involved, while another muscle does not. On the surface, this would appear to be a mirror effect. However, unless it can be shown that MEPs in the second muscle can be enhanced by observation of a different action (in which the second muscle is involved), it could be due to mechanisms distinct from those that generate mirror responses (e.g. if the TMS coil is placed closer to the motor representation of the first than the second muscle). Such a ‘two-action/two-muscle’ design, in which recordings are made from two muscles and two actions are presented, also permits the experimenter to rule out mirror-like responses that are not muscle specific (e.g. greater responses in both muscles to the observation of a particular action would imply a general motor response to that action rather than a muscle-specific, mirror response). In this two-action/two-muscle design, a true mirror effect is indicated by an interaction in MEP size between the muscle recorded and the action presented, indicating that muscle A responds more to the presentation of action X, in which it is involved, than to the presentation of action Y, in which it is not involved, whereas muscle B shows the opposite pattern of responses.

In summary, the data surveyed above and in Supplementary Data suggest that motor responses to action observation, including mirror neuron responses, first occur around 170–300 ms after action onset. However, this has not been investigated systematically using a technique that specifically measures *mirror* responses. The first aim of this study, therefore, was to use the two-action/two-muscle design to establish the timecourse of mirror effects. In Experiment 1, MEPs were recorded from the index (first dorsal interosseous, FDI) and little (abductor digiti minimi, ADM) finger abductor muscles during the observation of index and little finger abduction actions, at five timepoints between 100 and 300 ms after action onset.

### Counter-mirror effects

A number of studies using a range of methods have demonstrated that mirror responses can be abolished or reversed through ‘counter-mirror’ sensorimotor training, in which the sight of one action is paired with performance of a different action (Heyes *et al.*, 2005; Catmur *et al.*, 2007, 2008, 2011; Gillmeister *et al.*, 2008; Cook *et al.*, 2010, 2012; Wiggett *et al.*, 2011; see Catmur, 2013, for a review). Because a change in mirror responses is not observed after compatible training, in which participants perform the same movements as those they observe, these counter-mirror effects cannot be due to visual or motor experience alone, but must be due to the observation–execution contingency experienced during counter-mirror training (Catmur *et al.*, 2007). These results confirm the predictions of the associative account which suggests that mirror neurons’ sensorimotor matching properties are forged by sensorimotor experience (Heyes, 2001, 2010).

A recent article (Barchiesi and Cattaneo, 2012) queried whether these counter-mirror effects follow the same timecourse as mirror effects: in that study, effects of training on the direction of TMS-evoked movements were not found until 320 ms after observed action onset. The second aim of this study was therefore to investigate whether mirror and counter-mirror effects follow the same timecourse. In Experiment 2, MEPs were measured from the FDI and ADM muscles during observation of index and little finger actions before and after counter-mirror sensorimotor training, at those timepoints at which a significant mirror effect was found in Experiment 1.

## EXPERIMENT 1

### Method

#### Participants

Fourteen right-handed volunteers (seven women) aged 18–32 years (mean 23.8) took part. None had a history of neurological, major medical or psychiatric disorders. They had normal or corrected-to-normal visual acuity and were free from any contraindication to TMS (Wasserman, 1998; Rossi *et al.*, 2009). Before the study participants gave their written informed consent. They were naive as to the study purpose. The experimental procedures were approved by the local Ethics Committee and carried out in accordance with the principles of the revised Helsinki Declaration (World Medical Associations General Assembly, 2008). Participants were financially compensated for their time. None of the individuals taking part in the experiment experienced discomfort during TMS.

#### Electromyographic and TMS recording

TMS pulses were administered via a Magstim 200 stimulator (Magstim, Dyfed, UK) connected to a 70 mm figure-of-eight coil positioned over the left primary motor cortex (M1) hand region. The coil was held tangentially to the scalp with the handle pointing backward and laterally at 45° to the midline (Brasil-Neto *et al.*, 1992; Mills *et al.*, 1992). During the recording sessions, the coil was positioned at the optimal scalp position (OSP), defined as the position from which MEPs with maximal amplitude were recorded simultaneously from FDI (the muscle involved in index finger abduction) and ADM (the muscle involved in little finger abduction). To find the individual OSP, the coil was moved in steps of 1 cm over the motor cortex and the OSP was marked on a bathing cap worn by the participant. Once it was found, the individual resting motor threshold (rMT) was determined as the lowest stimulus intensity that induced at least 5 MEPs (of at least 50 μV of peak-to-peak amplitude) out of 10 consecutive TMS pulses in both muscles (Rossini *et al.*, 1994). Mean rMT was 45.2% (range 32–60%) of maximum stimulator intensity. During the recording sessions, stimulation intensity was set to 115% of rMT. MEPs were recorded simultaneously from FDI and ADM muscles of the participant’s right hand. The electromyographic (EMG) recording was performed through pairs of Ag–AgCl surface electrodes (10 mm diameter) placed over the muscle belly (active electrode) and over the associated joint or tendon (reference electrode). The ground was placed over the participant’s right wrist. The signal was sampled (5000 Hz), amplified, band-pass filtered (10–1000 Hz) with a 50-Hz notch filter and stored for off-line analysis. Data were collected from 100 ms before to 200 ms after the TMS pulse.

#### Stimuli

**Fig. 1:**
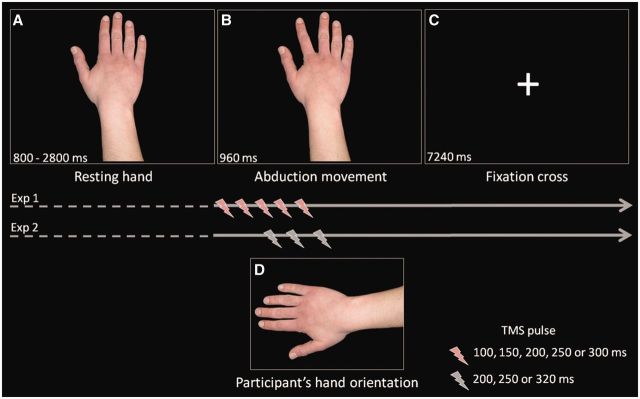
Example of experimental procedure for TMS sessions. A resting hand (**A**) was shown for a variable delay (from 800 to 2800 ms) in a prone position, vertically oriented. Following the resting hand, the endpoint of one of the two abduction actions (**B**, index abduction) was presented for 960 ms and was followed by a fixation cross (**C**) lasting 7240 ms. During the abduction action, the TMS pulse was delivered at one of five (Experiment 1) or three (Experiment 2) different timepoints after action onset. The participant’s right arm was placed in a horizontal orientation across their body (**D**) and was covered by a screen such that it was not visible to the participant.

The experimental stimuli comprised action sequences created from two static photographs of the dorsal view of the right hand of a female. An apparent motion effect was obtained by presenting single frames of a right hand at rest followed by the endpoint of either an index or little finger abduction. On each trial, the hand of the model was shown in a prone position, vertically oriented, with fingers toward the top of the screen. Following a variable delay (800–2800 ms) after presentation of the resting hand, the endpoint of one of the two abduction actions was presented for 960 ms (Figure 1), after which it was replaced by a white fixation cross on a black background for 7240 ms.

#### Procedure

Participants were seated in a comfortable chair in a dimly illuminated room. A chinrest was used to standardize viewing distance and to provide support. The participant’s right arm was placed in a horizontal orientation across their body, controlling for both simple and orthogonal spatial compatibility between the participant’s hand and the stimulus hand. The participant’s hand was covered by a screen such that it was not visible to the participant.

Participants were instructed to keep their right hand still and as relaxed as possible and to pay attention to the visual stimuli. To control for attention, participants were asked to watch out for a faint circle that appeared on the stimulus hand on 10% of trials. Four times per block, a question was presented on the monitor asking whether the circle was present on the previous trial. Responses were made with the left hand. The experiment comprised three blocks of 40 TMS trials. In each block, 20 index and 20 little finger abduction actions were presented in a randomized order. For each type of observed action, the TMS pulse was randomly delivered at one of five timepoints: 100, 150, 200, 250 and 300 ms after the onset of the frame depicting the endpoint of the action. A total of 12 TMS trials were administered for each cell of the design (two observed actions × five timepoints). Stimuli were presented and TMS pulses triggered using E-Prime (Psychology Software Tools, PA, USA).

#### Data analysis

In order to prevent contamination of MEP measurements by background EMG activity, trials with background activity greater than 100 µV in the 100 ms window preceding the TMS pulse were excluded from the MEP analysis. Peak-to-peak MEP amplitude was calculated for each muscle for each trial. MEP amplitudes less than 50 µV or deviating more than 2.5 s.d. from the mean for each muscle for each block were excluded as outliers. MEP amplitudes were normalized by dividing by the mean MEP amplitude for each muscle for each block.

### Results

**Fig. 2:**
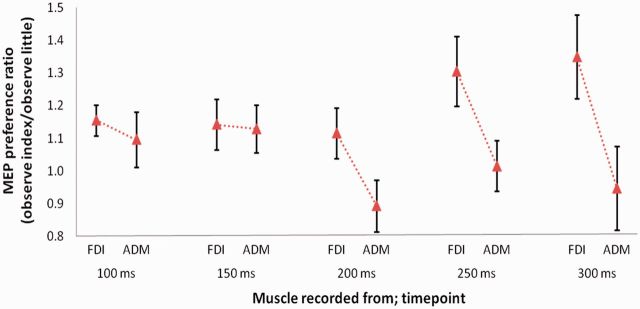
Experiment 1: Mean ± s.e.m. MEPs recorded from index and little finger muscles at five timepoints after observed action onset. For presentation purposes, MEP preference ratios are shown, calculated for each muscle as mean MEP size during observation of index finger actions divided by mean MEP size during observation of little finger actions. This ratio indicates the degree to which MEPs recorded in that muscle were greater for index than little finger action observation. A mirror effect is indicated by a higher value in the FDI (index finger muscle) than in the ADM (little finger muscle). All statistical analyses were applied to normalized MEP sizes. Significant mirror effects were found at 200, 250 and 300 ms.

The minimum number of MEPs in any cell was 10; an average of 11.7 ± 0.12 (s.d.) MEPs per cell were analysed. Raw MEP sizes are reported in Supplementary Table S1. For each muscle in every participant, mean normalized MEP sizes were calculated for each observation condition and TMS pulse timepoint (see Supplementary Table S2) and submitted to a 2 × 2 × 5 repeated-measures analysis of variance (ANOVA) with muscle (FDI and ADM), observed action (index and little finger abduction) and timepoint (100, 150, 200, 250 and 300 ms) as within-subjects factors. An interaction between muscle and observed action was obtained [*F*(1,13) = 6.903, *P* = 0.021], indicating a significant ‘mirror’ effect. However, the three-way interaction between muscle, observed action and timepoint was also statistically significant [*F*(4,52) = 2.804, *P* = 0.035], indicating that the mirror effect differed across timepoints. Simple interaction analyses were performed to test for the presence of a mirror effect (interaction between muscle and observed action) at each of the five timepoints. No mirror effect was obtained at timepoints of 100 and 150 ms after action onset; however, significant mirror effects were found for timepoints of 200, 250 and 300 ms [*F*(1,13) = 8.597, *P* = 0.012; *F*(1,13) = 5.381, *P* = 0.037; *F*(1,13) = 5.012, *P* = 0.043, respectively] illustrated in Figure 2. No other main effects or interactions reached significance.

## EXPERIMENT 2

### Method

A change of institution necessitated the use of different TMS equipment between Experiments 1 and 2. The number of trials per cell of the design was increased to 20 by stimulating only at the three timepoints where a significant mirror effect was found in Experiment 1. This shortened the TMS acquisition time sufficiently that baseline trials could also be included. All other procedures, except where stated, were identical to Experiment 1.

#### Participants

Eighteen new volunteers (11 women) aged 19–45 years (mean 26.1) took part.

#### EMG and TMS recording

TMS pulses were administered via a Magstim Rapid 2 stimulator (Magstim, Dyfed, UK). Mean rMT was 67.8% pre-training (range 49–82%) and 66.4% post-training (range 49–80%) of maximum stimulator intensity. The signal was band-pass filtered between 3 and 1000 Hz.

#### Stimuli

The experimental stimuli were identical to Experiment 1. The baseline stimulus comprised a white fixation cross on a black background, presented for a variable duration (8040–9640 ms).

#### Procedure

Experiment 2 comprised a pre-training TMS session, a counter-mirror sensorimotor training session and a post-training TMS session. The three sessions for Experiment 2 were administered on three different days with 24 h separating the training and post-training TMS sessions.

Each TMS session comprised four blocks of 40 TMS trials. In each block, 15 index and 15 little finger abduction actions, and 10 baseline trials, were presented in a randomized order. For each type of observed action, the TMS pulse was randomly delivered at one of the three timepoints: 200, 250 and 320 ms after the onset of the frame depicting the endpoint of the action. During baseline trials, the TMS pulse was delivered randomly between 800 and 2800 ms after trial onset.

During the counter-mirror sensorimotor training session, 12 blocks, each comprising 70 trials (35 index and 35 little finger actions in a randomized order), were presented. Trial structure was identical to the TMS sessions with the exception that the fixation cross was presented for 2000 ms after each action. Participants were instructed to respond by abducting their little finger whenever an index finger abduction was shown and abducting their index finger whenever a little finger abduction was shown. Participants were incentivized to respond as quickly and accurately as possible by receiving an extra £0.50 for each block in which their mean response time (RT) was below 400 ms and 4 or fewer errors were made. RTs were measured using EMG recording from the FDI and ADM muscles, as for the TMS sessions.

#### Data analysis

The baseline for each muscle for each block was calculated as the mean amplitude of MEPs recorded from that muscle during baseline trials. MEP amplitudes were normalized by dividing the baseline value. For the training session, RTs were calculated using the Brain Vision Analyzer ‘EMG Onset’ solution. This searches for the timepoint at which EMG activity exceeds 6 s.d. from the mean of the baseline period (200 ms before observed action onset).

### Results

#### Training session

**Fig. 3:**
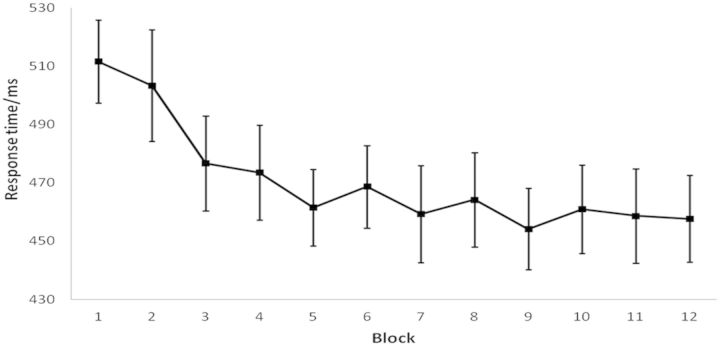
Experiment 2: mean ± s.e.m. response times during sensorimotor training.

Mean RT was calculated for each block (Figure 3) and submitted to a repeated-measures ANOVA with block (1–12) as the within-subjects factor. A main effect of block was observed [*F*(11,187) = 6.477, *P* < 0.001], suggesting that counter-mirror performance improved during training. This conclusion was supported by a significant linear decrease in RT across blocks [*F*(1,17) = 11.981, *P* = 0.003] and by the finding that RT for the final block was significantly lower than for the first block [*t*(17) = 3.880, *P* = 0.001].

#### MEP data

**Fig. 4:**
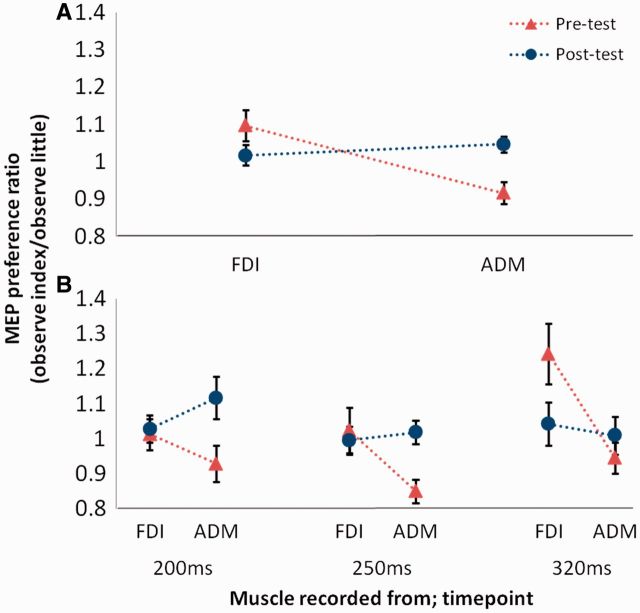
Experiment 2: mean ± s.e.m. MEPs recorded from index and little finger muscles before and after counter-mirror sensorimotor training, at three timepoints after observed action onset. MEP preference ratios are shown, where a higher value in the FDI than the ADM indicates a mirror effect, while the reverse pattern indicates a counter-mirror effect. A significant effect of training was found across all timepoints (**A**) and at each timepoint individually (**B**).

The minimum number of MEPs in any cell was 12; an average of 18.03 ± 2.51 (s.d.) (pre-training) and 18.39 ± 2.46 (post-training) MEPs per cell were analysed. Raw MEP sizes are reported in Supplementary Table S3. For each muscle in every participant for both pre- and post-training sessions, mean normalized MEP sizes were calculated for each observation condition and TMS pulse timepoint (see Supplementary Table S4) and submitted to a 2 × 2 × 2 × 3 repeated-measures ANOVA with session (pre-training and post-training), muscle (FDI and ADM), observed action (index and little finger) and timepoint (200, 250 and 320 ms) as within-subjects factors. A significant mirror effect (interaction between muscle and observed action) was observed [*F*(1,17) = 8.864, *P* = 0.008]. However, this effect was modulated by the factor of testing session, yielding a significant three-way interaction between session, muscle and observed action [*F*(1,17) = 23.617, *P* < 0.001], indicating that counter-mirror training altered the mirror effect (Figure 4A). Crucially, the four-way interaction between session, muscle, observed action and timepoint was not statistically significant [*F*(2,34) = 0.332, *P* = 0.720]. This result implies that the effect of counter-mirror training was the same at all three timepoints. Confirming this conclusion, simple interaction analyses revealed a three-way interaction (session × muscle × observed action) at each timepoint [200 ms: *F*(1,17) = 6.476, *P* = 0.021; 250 ms: *F*(1,17) = 10.212, *P* = 0.005; 320 ms: *F*(1,17) = 7.496, *P* = 0.014; see Figure 4B].

The only other main effect or interaction to reach significance was a main effect of session, indicating that MEP sizes during action observation were reduced relative to baseline in the post-training session [*F*(1,17) = 8.664, *P* = 0.009]. One possible explanation for this result is that, compared with the pre-training session, participants were more able to anticipate the delivery of the TMS pulse during action observation trials, *vs* baseline trials where the time window for TMS delivery was much larger. Support for this anticipatory account is presented in Supplementary Data.

## DISCUSSION

Using a two-action/two-muscle design, Experiment 1 demonstrated that mirror responses to observed actions (defined as an interaction between muscle and observed action; see ‘Introduction’ section) can be detected from around 200 ms after action onset. As in nearly all other human research on mirror neurons, our measure does not provide a direct measure of mirror neuron activity; however, this timepoint is consistent with the results obtained from single-cell macaque neurophysiology experiments. In humans, a similar timecourse of mirror responses has been previously suggested by MEG experiments; the nature of the observed response, however, remained unclear. The present method permits us to conclude that a *mirror*, rather than a more general motor, response to the observed action is present at this timepoint. In addition, by using simple actions generated via apparent motion, the timing of the mirror response was isolated in a way that is not possible with more naturalistic actions. The ongoing character of naturalistic actions means that measurement of the timecourse is often confounded with the extent of movement that has taken place. For example, MEPs measured at 100 and 300 ms after the onset of an observed reach–grasp action will differ not only in terms of whether information about the observed action has reached motor cortex by the time of the TMS pulse but also in terms of the amount of movement that has occurred, and the phase of the observed action at these timepoints. In that case, failure to find a mirror response at 100 ms after action onset could be because the relevant information has not yet reached motor cortex, or because the force requirement of the action (see Alaerts *et al.*, 2010) at that timepoint is not sufficient to produce a mirror response. The use of apparent motion avoids such a problem because the extent of movement, and thus the action phase, is the same at all timepoints after action onset. Thus, we consider the use of apparent motion to be crucial in isolating information about the timecourse of the response to observed actions.

The finding that mirror responses can be measured from around 200 ms after action onset has a number of implications. First, it suggests that previous reports of very early (<100 ms) ‘mirror’ responses to discrete, non-recurring actions are likely to be non-specific alerting or spatial effects. Similar early responses can be seen in the results of Experiment 1 at 100 and 150 ms; however, the lack of difference between the two muscles demonstrates that these are non-specific responses (both muscles respond equally to the observation of index finger actions), rather than mirror responses in which the specific motor programs necessary to perform the observed actions are activated. Such mirror responses, defined as an interaction between muscle and observed action, appear at 200 ms. (We speculate that the apparent strong response in the ADM at 200 ms in Figure 2 is due to this muscle no longer responding in a generic fashion to observation of index finger movements, but instead responding in a specific fashion that differentiates between the two observed actions. A differential response to the two observed actions is also present in the FDI at 200 ms, but this is not apparent when inspecting Figure 2 because of the generic response to index finger movements at the earlier timepoints.) The second implication relates to the possible functions of mirror responses in social cognition. If mirror responses occur around 200 ms after the onset of an observed action, rather than earlier, then this places some constraints on the types of function that mirror responses could contribute to. For example, it is less likely that mirror responses underlie the link between ‘fast motor resonance’ and empathy reported by Lepage *et al.* (2010). It will be important for future research to investigate the timecourse of mirror responses to more complex actions, as these may take longer to produce a mirror response; and to investigate the role played by prediction in modulating the timecourse of mirror responses to actions unfolding in more naturalistic settings.

Experiment 2 demonstrated a significant effect of counter-mirror training for all timepoints at which mirror responses were present in Experiment 1 (and prior to training in Experiment 2). This result suggests that mirror and counter-mirror effects share the same timecourse, supporting the possibility that the transformation of sensory to motor information during action observation occurs via a similar neuroanatomical pathway for both mirror and counter-mirror responses (see also Catmur *et al.*, 2011). Such a finding would confirm the predictions of the associative account (Heyes, 2001, 2010). If counter-mirror responses had been found to follow a slower timecourse, this might have suggested that such responses are the result of a more indirect route, for example via prefrontal areas for rule retrieval. [There is of course a possibility that counter-mirror training has its effects earlier than the earliest (200 ms) timepoint tested in Experiment 2; however, if this were the case it would be even less likely that these effects arise via an indirect route.] It is quite likely that such a route is involved during the early part of the training session when participants retrieve a rule in order to follow task instructions (e.g. ‘if index, do little’). However, the current results suggest that any such rule-based responding merely initiates associative learning and, after new counter-mirror associations between observed and performed actions have been formed and consolidated, subsequent action observation activates counter-mirror responses directly, via the same timecourse as mirror responses. It is also possible that, during training, participants learned to associate not only the identity but also the location of the observed action with the relevant response. Associative learning theory suggests that any aspect of a stimulus (including its spatial location) which has a predictive relationship with a response may form associations with that response, and thus this possibility is not in conflict with the predictions of the associative account.

In conclusion, this study has demonstrated that mirror responses can be measured from around 200 ms after observed action onset, and that effects of counter-mirror training follow the same timecourse. By demonstrating that mirror and counter-mirror responses take place over the same timescale, these results lend support to the suggestion that these responses involve similar neuroanatomical pathways and thus that mirror responses may originally arise from sensorimotor experience. In addition, by demonstrating the timecourse of mirror responses, these results provide an important reference point for the investigation of the functions of mirror responses in social cognition.

## SUPPLEMENTARY DATA

Supplementary data are available at *SCAN* online.

Supplementary Data
