# Supplementary material for: Timecourse of mirror and counter-mirror effects measured with transcranial magnetic stimulation
Source: Soc Cogn Affect Neurosci. 2013 May 23;9(8):1082–8. doi: 10.1093/scan/nst085 (PMC4127010; doi:10.1093/scan/nst085)
Supplement: Supplementary Data [file supp_9_8_1082__index.html]

Timecourse of mirror and counter-mirror effects measured with transcranial magnetic stimulation — Timecourse of mirror and counter-mirror effects measured with transcranial magnetic stimulation — Supplementary Data 

# Timecourse of mirror and counter-mirror effects measured with transcranial magnetic stimulation

## Supplementary Data

files

**Files in this Data Supplement:**

- Supplementary Data - docx file
