# Supplementary material for: Timecourse of mirror and counter-mirror effects measured with transcranial magnetic stimulation
Source: Soc Cogn Affect Neurosci. 2013 May 23;9(8):1082–8. doi: 10.1093/scan/nst085 (PMC4127010; doi:10.1093/scan/nst085)
Supplement: Supplementary Data [file supp_nst085_scan-12-337-File006.docx]

Supplementary Material: Timecourse of mirror and counter-mirror effects measured with transcranial magnetic stimulation

Andrea Cavallo, Cecilia Heyes, Cristina Becchio, Geoffrey Bird, & Caroline Catmur

**Supplementary literature review regarding timecourse of mirror neuron responses**

In order to assess the timecourse of mirror responses it is important to use discrete, non-recurring actions such that the time of onset of the action can be clearly determined, and the action cannot be predicted in advance of its onset. To the extent that these conditions are met in the macaque mirror neuron literature, it is possible to estimate the timecourse of mirror neuron responses from average latency information provided in that literature, or by measuring raster diagrams representing the discharge of single neurons in relation to action onset. For example, Gallese et al. (1996; Figure 1A) describe the responses of a single neuron during eight action observation trials. Across these trials, this neuron demonstrates a mean firing latency of around 730 ms after trial onset; however, it is unclear whether trial onset also represents the start of the experimenter’s action. The study by Kraskov et al. (2009) is more informative in this regard. Figures 2G and 2H illustrate the responses of a single neuron during ten action observation trials in which the onset of the experimenter’s action is also indicated. During action observation this neuron increases its firing relative to baseline, commencing at around 250 ms after the onset of the experimenter’s action. Figure 3A depicts the population responses of 11 facilitation and 14 suppression mirror neurons during action observation. Assuming the experimenter’s action was initiated at a similar time to those in Figure 2G, it can be deduced that the population response of facilitation mirror versus non-mirror neurons diverges at around 300 ms after action onset, with the response of suppression mirror neurons diverging slightly earlier.

When results are compared across the macaque mirror neuron literature, it is clear that the timecourse of responses to perceived actions in premotor area F5 varies widely, depending on the stimulus type and task demands. Early responses with a latency of around 170 ms to auditory action stimuli were recorded by Keysers and colleagues (2003); fast responses of around 200 and 250 ms to visual stimuli were also found by Bonini et al. (2009) and Umiltà et al. (2001) respectively. Rochat and colleagues (2010) reported that mirror neuron response latency varied with the observed action type (400 ms after hand opening; 680 ms after the onset of a stick movement; and 890 ms after the opening of pliers), and for actions presented via video, mirror neuron responses were as slow as 1800 ms (Caggiano et al., 2011). Thus the macaque mirror neuron literature does not currently provide a clear indication of how quickly mirror responses to others’ actions occur.

**Supplementary analysis, Experiment 2**

The main effect of session found in Experiment 2 indicated that MEP sizes during action observation were reduced relative to baseline in the post-training session (F_(1,17)_ = 8.664, *p* = .009). One possible explanation for this result is that, compared to the pre-training session, participants were more able to anticipate the delivery of the TMS pulse during action observation trials, versus baseline trials where the time window for TMS delivery was much larger. This would allow participants to activate inhibitory mechanisms in order to suppress any TMS-driven excitatory influence during action observation (Villiger et al., 2011). If this anticipatory account is correct, there should be a linear decrease in MEP size relative to baseline across the course of the whole experiment, as participants learn to anticipate TMS delivery on action observation trials. Mean normalised MEP sizes for both sessions were therefore entered into a repeated-measures ANOVA with within-subjects factor of block (1 to 8). A significant linear effect of block was found (F_(1,17)_ = 12.124, *p* = .003), suggesting that the main effect of session described above was the result of ongoing learning across the course of the whole experiment regarding the likely delivery time of the TMS pulse.

**Supplementary Tables**

**Supplementary Table 1.** Raw (in µV) mean (± SD) peak-to-peak MEP amplitudes recorded during Experiment 1. FDI, first dorsal interosseous; ADM, abductor digiti minimi.

| Muscle | MEP amplitude |
| --- | --- |
| FDI | 871 (494) |
| ADM | 434 (262) |

**Supplementary Table 2.** Normalised mean (± SD) MEP amplitudes recorded during Experiment 1. FDI, first dorsal interosseous; ADM, abductor digiti minimi.

|  |  | Timepoint | | | | |
| --- | --- | --- | --- | --- | --- | --- |
| Muscle | Observed movement | 100 ms | 150 ms | 200 ms | 250 ms | 300 ms |
| FDI | Index ()()()()()()()  Little | 0.967 (0.122)  0.875 (0.133) | 1.092 (0.229)  1.000 (0.155) | 1.090 (0.239)  1.023 (0.111) | 1.154 (0.165)  0.919 (0.203) | 1.068 (0.228)  0.822 (0.201) |
| ADM | Index ()()()()()  Little | 0.969 (0.222)  0.926 (0.221) | 1.030 (0.233)  0.955 (0.210) | 0.960 (0.280)  1.139 (0.224) | 1.006 (0.137)  1.042 (0.258) | 0.940 (0.218)  1.048 (0.372) |

**Supplementary Table 3.** Raw (in µV) mean (± SD) peak-to-peak MEP amplitudes recorded during Experiment 2. FDI, first dorsal interosseous; ADM, abductor digiti minimi.

| Muscle | Pre-training | Post-training |
| --- | --- | --- |
| FDI | 1177 (1228) | 1241 (952) |
| ADM | 241 (150) | 305 (215) |

**Supplementary Table 4.** Normalised mean (± SD) MEP amplitudes recorded during Experiment 2. FDI, first dorsal interosseous; ADM, abductor digiti minimi.

|  |  | Pre-training timepoint | | |  | Post-training timepoint | | |  |
| --- | --- | --- | --- | --- | --- | --- | --- | --- | --- |
| Muscle | Observed movement | 200 ms | 250 ms | 320 ms |  | 200 ms | 250 ms | 320 ms |  |
| FDI | Index ()()()()()()()  Little | 0.949 (0.131)  0.938 (0.114) | 0.970 (0.184)  0.948 (0.289) | 1.099 (0.188)  0.885 (0.169) |  | 0.834 (0.102)  0.811 (0.153) | 0.912 (0.136)  0.917 (0.190) | 0.856 (0.143)  0.822 (0.179) |  |
| ADM | Index ()()()()()  Little | 0.938 (0.158)  1.011 (0.271) | 0.918 (0.148)  1.081 (0.199) | 0.950 (0.154)  1.005 (0.199) |  | 0.934 (0.111)  0.836 (0.186) | 0.961 (0.147)  0.944 (0.136) | 0.944 (0.168)  0.935 (0.217) |  |

**Supplementary References**

Bonini, L., Rozzi, S., Serventi, F.U., Simone, L., Ferrari, P.F., Fogassi, L. (2009). Ventral premotor and inferior parietal cortices make distinct contribution to action organization and intention understanding. *Cerebral Cortex*, *20*, 1372-85.

Caggiano, V., Fogassi, L., Rizzolatti, G., Pomper, J.K., Thier, P., Giese, M.A., Casile, A. (2011). View-based encoding of actions in mirror neurons of area f5 in macaque premotor cortex. *Current Biology*, *21*, 144-8.

Gallese, V., Fadiga, L., Fogassi, L., Rizzolatti, G. (1996). Action recognition in the premotor cortex. *Brain, 119,* 593-609.

Keysers, C., Kohler, E., Umiltà, M.A., Nanetti, L., Fogassi, L., Gallese, V. (2003). Audiovisual mirror neurons and action recognition. *Experimental Brain Research*, *153*, 628-36.

Kraskov, A., Dancause, N., Quallo, M.M., Shepherd, S., Lemon, R.N. (2009). Corticospinal neurons in macaque ventral premotor cortex with mirror properties: a potential mechanism for action suppression? *Neuron*, *64*, 922-30.

Rochat, M.J., Caruana, F., Jezzini, A., Escola, L., Intskirveli, I., Grammont, F., Gallese, V., Rizzolatti, G., Umiltà, M.A. (2010). Responses of mirror neurons in area F5 to hand and tool grasping observation. *Experimental Brain Research*, *204*, 605-16.

Umiltà, M.A., Kohler, E., Gallese, V., Fogassi, L., Fadiga, L., Keysers, C., Rizzolatti, G. (2001). I know what you are doing: a neurophysiological study. *Neuron*, *31*, 155-65.

Villiger, M., Chandrasekharan, S., Welsh, T.N. (2011). Activity of human motor system during action observation is modulated by object presence. *Experimental Brain Research, 209,* 85-93.
